# Chemical consequences of cutaneous photoageing

**DOI:** 10.1186/1752-153X-6-34

**Published:** 2012-04-25

**Authors:** Sarah A Thurstan, Neil K Gibbs, Abigail K Langton, Christopher EM Griffiths, Rachel EB Watson, Michael J Sherratt

**Affiliations:** 1 Inflammation Sciences, The University of Manchester, Manchester Academic Health Science Centre, Manchester, UK; 2 Developmental Biomedicine Research Groups, The University of Manchester, Manchester Academic Health Science Centre, Manchester, UK

## Abstract

Human skin, in common with other organs, ages as a consequence of the passage of time, but in areas exposed to solar ultraviolet radiation, the effects of this intrinsic ageing process are exacerbated. In particular, both the severity and speed of onset of age-related changes, such as wrinkle formation and loss of elasticity, are enhanced in photoaged (also termed extrinsically aged) as compared with aged, photoprotected, skin. The anatomy of skin is characterised by two major layers: an outer, avascular, yet highly cellular and dynamic epidermis and an underlying vascularised, comparatively static and cell-poor, dermis. The structural consequences of photoageing are mainly evident in the extracellular matrix-rich but cell-poor dermis where key extracellular matrix proteins are particularly susceptible to photodamage. Most investigations to date have concentrated on the cell as both a target for and mediator of, ultraviolet radiation-induced photoageing. As the main effectors of dermal remodelling produced by cells (extracellular proteases) generally have low substrate specificity, we recently suggested that the differential susceptibility of key extracellular matrix proteins to the processes of photoageing may be due to direct, as opposed to cell-mediated, photodamage.

In this review, we discuss the experimental evidence for ultraviolet radiation (and related reactive oxygen species)-mediated differential degradation of normally long lived dermal proteins including the fibrillar collagens, elastic fibre components, glycoproteins and proteoglycans. Whilst these components exhibit highly diverse primary and hence macro- and supra-molecular structures, we present evidence that amino acid composition alone may be a useful predictor of age-related protein degradation in both photoexposed and, as a consequence of differential oxidation sensitivity, photoprotected, tissues.

## Introduction

Human skin undergoes extensive changes in appearance (e.g. wrinkle formation) and mechanical function (loss of both compliance and resilience) with age [1-3]. Whilst these structural and functional changes eventually manifest in elderly, photoprotected skin, their age of onset is accelerated and their severity is exacerbated by exposure to environmental factors such as smoking and ultraviolet radiation (UVR) [4-6]. Exposure to UVR, in particular, induces extensive changes in the composition and architecture of the extracellular matrix (ECM)-rich dermis [7,8]. Although UVR undoubtedly influences the viability and phenotype of cutaneous cells, the ability of these cells to selectively remodel key elements of the ECM via production of low substrate specificity proteases may be limited [9]. In this review, we discuss: i) the composition of healthy skin: ii) the effects of UVR exposure on skin structure and function, iii) experimental evidence that UVR directly and differentially degrades skin biomolecules and: iv) the potential for amino acid composition alone (as opposed to higher order structures) to predict the susceptibility of key ECM proteins to direct (via UVR absorption) and indirect (via photodynamically produced reactive oxygen species [ROS]) degradation.

## Structure and function of young, healthy skin

Skin is divided into two regions: an external epidermis and internal dermis, which differ profoundly in structure and hence function. The largely cellular epidermis acts as a barrier which blocks and/or mediates the passage of water, pathogens, heat and UVR [10,11]. In order to perform these functions, keratinocyte stem cells at the base of the epidermis undergo mitotic division to produce a supply of sequentially differentiating daughter keratinocytes which are ultimately shed a few weeks later as keratin-rich enucleated cells in a process known as desquamation [12].

**Figure 1:**
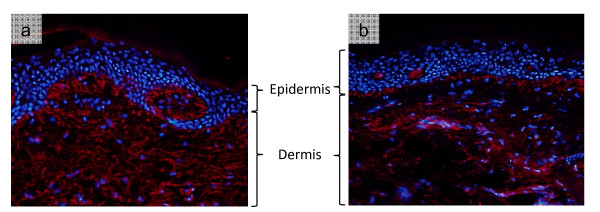
**The structure of skin is dominated by a highly cellular epidermis and a relatively acellular dermis.** Photoprotected **(a)** and photoaged **(b)** skin biopsies collected from the buttock and forearm respectively, of a 75 year old individual were immunofluorescently stained for the key elastic fibre component fibrillin-1 using a primary monoclonal antibody (clone 11C1.3) and a red fluorescently-labelled secondary antibody. Cellular DNA was visualised with diamidino-2-phenylindole (DAPI) which stains cell nuclei with blue fluorescence. In both photoprotected and photoexposed skin the cellular population is concentrated in the epidermis whilst in the dermis, fibroblasts are sparsely distributed. As a consequence, marked ECM remodelling in photoexposed skin (in this case loss of the red fibrillin-1 fluorescence) may be spatially separated from cells and hence from potential cell-derived mediators of tissue homeostasis.

In contrast to the dynamic epidermis, the structure of the dermis is characterised by a low density of fibroblast cells and a relatively static ECM [13]. Unlike intracellular proteins, which have half-lives measured in days, ECM proteins in human tissue are required to fulfil their mechanical and biochemical functions over a time course of many years in the absence of mechanisms to prevent or repair accumulated damage [14-17]. These proteins include members of the collagen super-family whose structures are characterised by the presence of at least one Gly-X-Y repeat domain (where X and Y are frequently proline and hydroxyproline amino acid residues respectively) which is able to form homo- or hetero-typic triple helices [18,19]. Although all collagens share a triple helical region, these otherwise structurally diverse proteins perform distinct and disparate mechanical roles. The network and anchoring collagens IV and VII for example are localised at the dermal-epidermal junction (DEJ) where they play key roles in binding the tissue layers together [20,21]. In contrast, the widely distributed fibrillar collagens I and III, form covalently bonded fibrils which resist tensile forces [22-24]. In order to withstand compressive forces human skin relies on hydrophilic glycosaminglycans (GAGs) including dermatan, chondroitin, heparin and keratin sulphate [25,26]. With the exception of hyaluronic acid, these un-branched disaccharide oligomers are located on post-translationally glycosylated proteins (proteoglycans) such as aggrecan, decorin and versican [27,28]. Finally, and in common with tissues of the cardiovascular and pulmonary systems, which are subjected to cyclic loads, human skin is rich in elastic fibres which drive passive recoil [29,30]. In young healthy skin, the architecture and relative abundance of the two major components of this system: the cross-linked, hydrophobic and highly compliant elastin core and the outer mantle of biochemically active and potentially mechanically stiff fibrillin-rich microfibrils is precisely controlled [31-33]. It is this elastic fibre system, and in particular the microfibrillar fraction which appears to be most sensitive to the effects of photoageing [9,34,35] (Figure 1).

## Ageing skin

All tissues in the human body exhibit some manifestations of intrinsic (chronological) ageing. Aged, yet photoprotected skin is characterised by a late onset of fine wrinkles, increased fragility and stiffness and by decreased elastic recoil [2,36,37]. These functional changes are correlated with structural remodelling (flattening) of the DEJ, a decrease in fibroblast numbers and dermal thickness and a generalised atrophy of dermal collagens, proteoglycans and elastic fibre components [38-41]. Although a consensus is yet to be achieved as to which cellular and molecular mechanisms play central causative roles in mediating intrinsic ageing: cell senescence, telomere shortening and oxidative stress have all been implicated in the process [42-46].

Compared with the slow, generalised atrophy of uncertain causation which characterises intrinsic skin ageing, extrinsically aged skin is characterised by a rapid and differential remodelling of diverse ECM components which is thought to be driven primarily by cellular responses to UVR [5,47]. Specifically, UVA radiation (315-400nm), which penetrates to a greater depth than UVB radiation (280-315nm), may be primarily responsible for chronic photoageing [5,48,49]. Clinically, photoaged skin appears deeply wrinkled and mottled and is characterised by reduced compliance and recoil [2,8]. Histologically these gross functional and structural differences are associated with: i) the loss of fibrillar collagens from the dermis as a whole and specifically with the localised loss of the elastic fibre associated proteins fibrillin (Figure 1) and fibulin-5 from the papillary dermis and: ii) the accumulation and often co-localisation of disorganised elastotic (elastic fibre containing) material and GAGs such as hyaluronic acid and chondroitin sulphate in a process known as solar elastosis [4,6,8,35].

**Figure 2:**
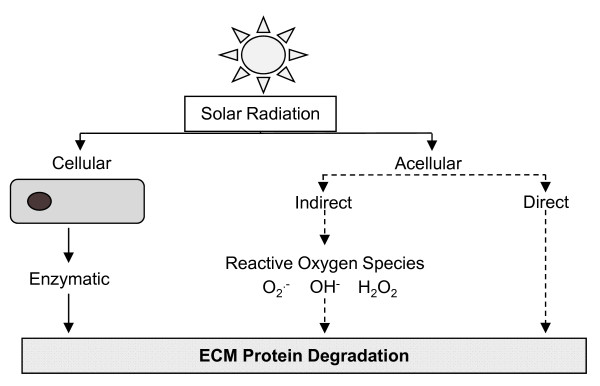
**Potential pathways of UVR-induced protein degradation.** Following exposure to UVR radiation, ECM remodelling in human skin may occur as a result of: i) cell mediated mechanisms via the synthesis of ECM proteases such as MMPs or ii) acellular pathways. Whilst cellular mechanisms undoubtedly play a role in downstream ECM remodelling, we recently demonstrated that physiologically attainable doses of UVR are capable of differentially degrading key ECM components in a cell-free environment. It remains to be determined whether this protein degradation occurs as a consequence of *direct* photon absorption by amino acid residues or the *indirect* action of UVR-induced ROS.

To date, attention has focussed primarily on UVR-mediated activation and up-regulation of proteolytic enzymes and in particular the matrix metalloproteinases (MMPs) as the key causative mechanism of ECM degradation in photoaged skin [47,50,51]. Collectively, however, the implicated MMPs (−1, -2, -3, -7, -8, -9, -12 and −13) are capable of degrading most dermal ECM components [9,52]. We therefore recently suggested that acellular (i.e. direct UVR interaction with ECM proteins), rather than cellular (UVR mediated synthesis of ECM proteases) mechanisms may be responsible for the selective degradation of elastic fibre associated glycoproteins in early photoageing (Figure 2).

## Degradation of biomolecules by UVR

Irrespective of whether direct UVR/molecule interactions alone or downstream perturbations in cell-mediated homeostasis are primarily responsible for photoageing, the process will be initiated by the absorption of photon energy by endogenous chromophores in the skin (Grotthuss–Draper law). Whilst, by definition, the term chromophore should be used only to refer to molecular regions which absorb visible or UV radiation, in many cases entire molecules or even molecular families are often referred to as UVR chromophores [53]. On absorption of photon energy, chromophores within ECM proteins enter a highly energetic but short lived, singlet excited state which may result in direct perturbations to protein structure [54]. In turn, this singlet state may undergo intersystem crossing to yield the longer lived triplet state which can act as an intra-molecular photosensitiser. Such photosensitisers are capable of undergoing type I (electron transfer) reactions to form radical species and/or type II (energy transfer) reactions with molecular O_2,_ resulting in the formation of singlet O_2_ (^1^O_2_) which is a major photo-oxidiser of other protein moieties [55,56]. As the main ECM chromophores (see following sections) primarily absorb in the UVB (280-315nm) region it is this waveband that is mainly responsible for ECM damage via the ECM singlet state and intra-molecular photosensitisation [54]. Whilst some collagen photosensitised production of ROS has been reported to occur on absorption of UVA (315-400nm) radiation, it is unclear whether an intra-molecular ECM chromophore or an endogenous chromophore (e.g. pyridinoline) was responsible [57]. Photosensitisation by extra-molecular chromophores (i.e. non-ECM chromophores associated with ECM proteins such as Advanced Glycation End products (AGEs)) is likely to play a major role in UVA-induced, and primarily ^1^O_2_–mediated, photo-oxidative ECM protein damage *in vivo*[57]. However, as the exact nature of these ECM-associated, extra-molecular photosensitisers is yet to be established, this is an exciting area of current research.

### Intracellular and epidermal chromophores

Predominant amongst skin chromophores are DNA, melanin, urocanic acid and proteins which have wavelength/photon energy specific absorption spectra (usually maximal in the UVB part of the solar UVR spectrum) [54]. Absorption of UVR by DNA results in the formation of photoproducts, including highly mutagenic cyclopyrimidine dimers, which if formed in crucial tumor suppressor genes (e.g. p53) and/or oncogenes (e.g. ras) may initiate skin tumorigenesis [58]. Melanin produced by epidermal melanocytes, absorbs UVR and acts as a natural sunscreen, protecting DNA and proteins of the basal layer cells, particularly stem cells. Urocanic acid (UCA) which is produced in the upper epidermal cell layers, also has a sunscreening role but paradoxically, absorption of UVR results in the production of a photoisomer (*cis*-UCA) which has immunosuppressive properties and may increase the progression of skin cancers [59].

### Dermal extracellular chromophores

UVR absorbing epidermal molecules such as melanin and UCA appear to play a protective role in absorbing UVR and both DNA and short-lived intracellular proteins are, at least partially, protected from the long term effects of UV-mediated damage by endogenous mechanisms which detect and repair or rapidly replace defective molecules [15,46,60]. In contrast, individual dermal ECM proteins and supra-molecular assemblies must continue to function in potentially harmful environments for many years [61]. For example, aspartic acid racemisation methods estimate the half-life of human dermal collagen as 15 years whilst pulmonary elastic fibre components are retained for the lifetime of the individual [16,62]. Such extended molecular life-spans provide ample opportunity for the accumulation of damage via external influences such as UVR [14,63]. Type I collagen for example, may be fragmented and rendered less thermally stable by exposure UVR, whilst hydrolysed and irradiated elastin undergoes extensive photodegradation [64-67]. Similar UVR exposure can also affect key molecular functions including collagen fibril assembly and protease susceptibility [65,68,69]. Crucially however, in order to influence the structure and biological function of isolated type I collagen and elastin, these studies employed either supra-physiological UVR doses (measured in J/cm^2^ as compared to the 50 mJ/cm^2^ required to induce minimal erythemal [47]) and/or the use of sources emitting non-physiologically relevant wavelengths (i.e. UVC radiation <280nm which is not a component of solar UVR) .

In contrast to these studies, we recently demonstrated that exposure to a UVB radiation dose of 50 mJ/cm^2^ had no detectable affect on the electrophoretic mobility (in both denaturing and native conditions) of monomeric type I collagen [9]. In the same study, we established that fibrillin microfibrils extracted from the elastic fibre system, undergo extensive and apparently stochastic ultrastructural modification following exposure to doses of UVB radiation as low as 20mJ/cm^2^, whilst a dose of 100mJ/cm^2^ is sufficient to induce aggregation of dimeric fibronectin. We suggested therefore that the differential susceptibility exhibited by these key dermal ECM components to UVR exposure *in vitro* may: i) explain the selective degradation of elements of the elastic fibre system (fibrillin-1 and fibulin-5) *in vivo* and ii) be mediated by their relative amino acid (and hence Similar UVR in title chromophore) composition.

## Amino acid composition as a predictor of UVR susceptibility

Relative UVR absorption is determined by molecular structure. Therefore, for highly heterogeneous polymeric molecules, the potential consequences of UV irradiation are likely to differ between molecular species. In the case of proteins only a subset of amino acid residues: cysteine, histidine, phenylalanine, tryptophan and tyrosine act as potent UV chromophores for solar UVR (280-400nm) [70]. Hence the relative UVR susceptibility of individual proteins may be mediated primarily by their differential amino acid composition (Table 1).

**Table 1:** **Relative amino acid composition of three key dermal ECM proteins.** Monomeric type I collagen ([α1(I)]_2_α2(I), accession numbers P02452 (α1) and P08123 (α2)) is rich in Gly and Pro but contains few UV-B chromophores (Cys, His, Phe, Trp and Tyr). In contrast, fibronectin, (accession number P02751) and in particular fibrillin-1 (accession number P35555), are rich in UV-B chromophores and in the case of Fibrillin-1 Cys residues [9].

Amino Acid	Collagen I (%)	Fibronectin (%)	Fibrillin-1 (%)
Ala (A)	11.2	4.0	3.2
Arg (R)	5.1	5.2	4.5
Asn (n)	1.5	4.2	6.6
Asp (D)	2.8	5.1	6.0
Cys (C)	0.0	2.6	12.7
Gln (Q)	2.6	5.5	3.6
Glu (E)	4.6	5.9	7.0
Gly (G)	33.1	8.2	10.7
His (H)	0.6	2.2	1.7
IIe (I)	1.0	4.7	5.2
Leu (L)	2.4	5.2	4.8
Lys (K)	3.4	3.2	3.9
Met (M)	0.6	1.1	1.8
Phe (F)	1.3	2.2	2.9
Pro (P)	21.8	7.9	6.2
Ser (S)	3.5	8.0	6.0
Thr (T)	1.7	10.8	5.8
Trp (W)	0.0	1.7	0.5
Tyr (Y)	0.3	4.2	3.3
Val (V)	2.6	8.0	3.8
Total chromophore content (%)	2.2	12.9	21.1

### Amino acids residues (Cys/Cys-Cys, His, Phe, Trp, Tyr)

Whilst the absorption peaks of these amino acids lie in the UVC (<280nm) region, which is not part of terrestrial solar radiation, all have absorption tails that all have absorption tails that extend into the UVB and UVA regions. The rank order of absorption at the longer wavebands is Trp > Tyr > Phe > Cys > His which, in combination with their relative susceptibility to oxidation, is an important factor when considering their relative contribution to protein photodegradation (reviewed in [54]). The complex photochemistry that follows excitation of these amino acids has been studied using steady state and time-resolved techniques [71]. Illustrative of these photo-processes are those observed for Trp and indeed, fluorescence from the Trp singlet state is predominant in proteins containing this amino acid. Intersystem crossing competes with this process to generate the Trp triplet which in turn photosensitises the production of ROS; O_2_ radicals by electron transfer or ^1^O_2_ by energy transfer [54]. The quenching of ^1^O_2_ by Trp, His, Tyr, Met, and Cys side-chains can result in a number of modifications to ECM-protein structure and therefore function (reviewed in [56]). Many structural proteins in the ECM are stabilised by intra-chain disulphide bonds which may be photodegraded directly or as a consequence of a radical cascade initiated by electron transfer from nearby Trp or Tyr residues [63].

### Differential amino acid composition of key dermal proteins

**Figure 3:**
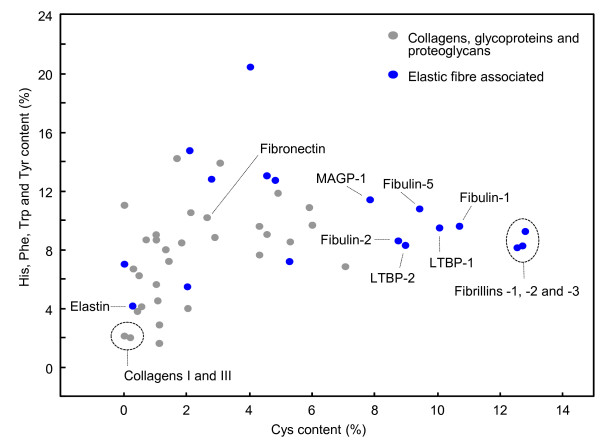
**Differential amino acid composition of major dermal ECM components.** Compared with the non-elastic fibre associated proteins (collagen: I, III, IV, V, VI, VII, VIII, XII, XIII, XIV, XVI, XVII, XXII and XXIII; the proteoglycans: fibromodulin, decorin, biglycan, perlecan, agrin, versican and aggrecan; and the glycoproteins: thrombospondin-1 and −2, tenascin-C and –X, osteopontin, fibronectin, laminin-5 and −6, vitronectin) elastic fibre components in general (MAGP-1 and −2, LTBP-1 and −2, MFAP-1, elastin, LOX, LOXL1, 2, 3 and 4, Fibulin-1, -2 and −3, emilin-1 and EBP) and the fibrillins in particular, are enriched in Cys residues. Furthermore, most of these in latter proteins are associated with the disulphide bonded microfibrils which are: i) degraded in the papillary dermis of mildly photoaged skin and ii) abundantly distributed in the elastotic material which characterises the deeper dermis of severely photoaged skin [34,74]. In contrast, the major structural components: dermal fibrillar collagen and elastin are almost devoid of UVR sensitive amino acids (Cys and His, Phe, Trp and Tyr residues).

The early and specific degradation of fibrillin-rich microfibrils and fibulin-5 from the photoexposed papillary dermis suggests that these ECM components may share structural similarities which pre-dispose them to UVR-mediated degradation [34,35]. The tertiary structures of many elastic fibre associated proteins, including fibrillins 1–3, fibulins 1–5 and the latent transforming growth factor β binding proteins (LTBPs) 1–4, are dominated by heavily disulphide bonded, calcium-binding epidermal growth factor (cbEGF)-like domains [72]. We suggested therefore, that the unequal distribution of UV chromophores and in particular Cys residues may explain both the differential degradation of fibrillin-rich microfibrils and fibronectin (but not collagen I or elastin) *in vitro* and the loss of fibrillin-rich microfibrils and fibulin-5 in the upper dermis following exposure to UVR [9,34,35]. Analysis of amino acid composition may therefore indicate which proteins are most likely to undergo direct UVR or, photodynamic and hence ROS-mediated degradation (Figure 3) [73].

## Conclusions

Skin presents an ideal model system in which to study the effects of ageing, whether due to the passage of time alone, or to the action of exogenous accelerating factors. Although the extensive structural remodelling which characterises the ageing process has profound consequences for cutaneous function, the primary causative mechanisms remain to be determined. We have discussed the evidence that selective UVR/molecule interactions alone may be sufficient to drive many of the characteristic remodelling events in photoaged skin.

## References

[B1] Agache PG Monneur C Leveque JL Derigal J Mechanical-properties and youngs modulus of human-skin in vivo Arch Dermatol Res 1980 269 221 232 10.1007/BF00406415 7235730

[B2] Escoffier C de Rigal J Rochefort A Vasselet R Leveque JL Agache PG Age-related mechanical properties of human skin: an in vivo study J Invest Dermatol 1989 93 353 357 10.1111/1523-1747.ep12280259 2768836

[B3] Langton AK Sherratt MJ Griffiths CEM Watson REB Review Article: A new wrinkle on old skin: the role of elastic fibres in skin ageing Int J Cosmet Sci 2010 32 330 339 10.1111/j.1468-2494.2010.00574.x 20572890

[B4] Tsoureli-Nikita E Watson REB Griffiths CEM Photoageing: the darker side of the sun Photochem Photobiol Sci 2006 5 160 164 10.1039/b507492d 16465300

[B5] Yaar M Gilchrest BA Photoageing: mechanism, prevention and therapy Br J Dermatol 2007 157 874 887 10.1111/j.1365-2133.2007.08108.x 17711532

[B6] Naylor EC Watson REB Sherratt MJ Molecular aspects of skin ageing Maturitas 2011 69 249 256 10.1016/j.maturitas.2011.04.011 21612880

[B7] Montagna W Kirchner S Carlisle K Histology of sun-damaged human-skin J Am Acad Dermatol 1989 21 907 918 10.1016/S0190-9622(89)70276-0 2808826

[B8] Warren R Gartstein V Kligman AM Montagna W Allendorf RA Ridder GM Age, sunlight, and facial skin: a histologic and quantitative study J Am Acad Dermatol 1991 25 751 760 10.1016/S0190-9622(08)80964-4 1802896

[B9] Sherratt MJ Bayley CP Reilly SM Gibbs NK Griffiths CEM Watson REB Low-dose ultraviolet radiation selectively degrades chromophore-rich extracellular matrix components J Pathol 2010 222 32 40 20552716 10.1002/path.2730

[B10] Blank IH Factors which influence the water content of the stratum corneum J Invest Dermatol 1952 18 433 440 14938659 10.1038/jid.1952.52

[B11] Proksch E Brandner JM Jensen J-M The skin: an indispensable barrier Exp Dermatol 2008 17 1063 1072 10.1111/j.1600-0625.2008.00786.x 19043850

[B12] Baker H Blair CP Cell replacement in the human stratum corneum in old age Br J Dermatol 1968 80 367 372 10.1111/j.1365-2133.1968.tb12322.x 11396507

[B13] Vitellarozuccarello L Cappelletti S Rossi VD Sarigorla M Stereological analysis of collagen and elastic fibers in the normal human dermis - variability with age, sex, and body region Anat Rec 1994 238 153 162 10.1002/ar.1092380202 8154602

[B14] Bailey AJ Molecular mechanisms of ageing in connective tissues Mech Ageing Dev 2001 122 735 755 10.1016/S0047-6374(01)00225-1 11322995

[B15] Jennissen HP Ubiquitin and the enigma of intracellular protein-degradation Eur J Biochem 1995 231 1 30 10.1111/j.1432-1033.1995.tb20665.x 7628459

[B16] Shapiro SD Endicott SK Province MA Pierce JA Campbell EJ Marked longevity of human lung parenchymal elastic fibers deduced from prevalence of D-aspartate and nuclear-weapons related radiocarbon J Clin Invest 1991 87 1828 1834 10.1172/JCI115204 2022748 PMC295305

[B17] Verzijl N DeGroot J Oldehinkel E Bank RA Thorpe SR Baynes JW Bayliss MT Bijlsma JWJ Lafeber F TeKoppele JM Age-related accumulation of Maillard reaction products in human articular cartilage collagen Biochem J 2000 350 381 387 10.1042/0264-6021:3500381 10947951 PMC1221264

[B18] Van Der Rest M Garrone R Collagen familiy of proteins FASEB J 1991 5 2814 2823 1916105

[B19] Birk DE Bruckner P Collagen suprastructures Collagen. Volume 247 2005 Springer-Verlag Berlin, Berlin 185 205 Topics in Current Chemistry]

[B20] Fleischmajer R Utani A MacDonald ED Perlish JS Pan TC Chu ML Nomizu M Ninomiya Y Yamada Y Initiation of skin basement membrane formation at the epidermo-dermal interface involves assembly of laminins through binding to cell membrane receptors J Cell Sci 1998 111 1929 1940 9645941 10.1242/jcs.111.14.1929

[B21] Brucknertuderman L Mitsuhashi Y Schnyder UW Bruckner P Anchoring fibrils and type-VII collagen are absent from skin in sever recessive dystrophic epidermolysis bullosa J Invest Dermatol 1989 93 3 9 10.1111/1523-1747.ep12277331 2746005

[B22] Henkel W Glanville RW Covalent crosslinking between molecules of type-I and type-III collagen - the involvement of the N-terminal, non-helical regions of the alpha-1(I) and alpha-1(III) chains in the formation of intermolecular crosslinks Eur J Biochem 1982 122 205 213 10.1111/j.1432-1033.1982.tb05868.x 6120835

[B23] Vogel HG Correlation Between Tensile Strength and Collagen Content in Rat Skin. Effect of Age and Cortisol Treatment Connect Tissue Res 1974 2 177 182 10.3109/03008207409152242 4279799

[B24] Gosline J Lillie M Carrington E Guerette P Ortlepp C Savage K Elastic Proteins: Biological Roles and Mechanical Properties Phil Trans Soc Lond B Biol Sci 2002 357 121 132 10.1098/rstb.2001.1022 PMC1692928 11911769

[B25] Waller JM Maibach HI Age and skin structure and function, a quantitative approach (II): protein, glycosaminoglycan, water, and lipid content and structure Skin Res Technol 2006 12 145 154 10.1111/j.0909-752X.2006.00146.x 16827688

[B26] Ruoslahti E Structure and biology of proteoglycans Annu Rev Cell Biol 1988 4 229 255 10.1146/annurev.cb.04.110188.001305 3143379

[B27] Danielson KG Baribault H Holmes DF Graham H Kadler KE Iozzo RV Targeted disruption of decorin leads to abnormal collagen fibril morphology and skin fragility J Cell Biol 1997 136 729 743 10.1083/jcb.136.3.729 9024701 PMC2134287

[B28] Zimmermann DR Dourszimmerman MT Brucknertuderman L Schubert M Versican is expressed in the proliferating zone in the epidermis and in association with the elastic network of the dermis J Cell Biol 1994 124 817 825 10.1083/jcb.124.5.817 8120102 PMC2119961

[B29] Cleary EG Gibson MA Elastin-associated microfibrils and microfibrillar proteins Int Rev Connect Tissue Res 1983 10 97 209 6358100 10.1016/b978-0-12-363710-9.50009-5

[B30] Kielty CM Stephan S Sherratt MJ Williamson M Shuttleworth CA Applying elastic fibre biology in vascular tissue engineering Phil Trans Roy Soc Lond B Biol Sci 2007 362 1293 1312 10.1098/rstb.2007.2134 17588872 PMC2440413

[B31] Cotta-pereira G Rodrigo FG Bittencourtsampaio S Oxytalan, elaunin, and elastic fibres in human skin J Invest Dermatol 1976 66 143 148 10.1111/1523-1747.ep12481882 1249442

[B32] Braverman IM Fonferko E Studies in cutaneous aging: I. The elastic fiber network J Invest Dermatol 1982 78 434 443 10.1111/1523-1747.ep12507866 7069221

[B33] Dahlback K Ljungquist A Lofberg H Dahlback B Engvall E Sakai LY Fibrillin immunoreactive fibers constitute a unique network in the human dermis: Immunohistochemical comparison of the distributions of fibrillin, vitronectin, amyloid P component, and orcein stainable structures in normal skin and elastosis J Invest Dermatol 1990 94 284 291 10.1111/1523-1747.ep12874430 1689758

[B34] Watson REB Craven NM Kang SW Jones CJP Kielty CM Griffiths CEM A short-term screening protocol, using fibrillin-1 as a reporter molecule, for photoaging repair agents J Invest Dermatol 2001 116 672 678 10.1046/j.1523-1747.2001.01322.x 11348454

[B35] Kadoya K Sasaki T Kostka G Timpl R Matsuzaki K Kumagai N Sakai LY Nishiyama T Amano S Fibulin-5 deposition in human skin: decrease with ageing and ultraviolet B exposure and increase in solar elastosis Br J Dermatol 2005 153 607 612 10.1111/j.1365-2133.2005.06716.x 16120151

[B36] Montagna W Carlisle K Structural changes in aging human skin J Invest Dermatol 1979 73 47 53 10.1111/1523-1747.ep12532761 448177

[B37] Smalls LK Wickett RR Visscher MO Effect of dermal thickness, tissue composition, and body site on skin biomechanical properties Skin Res Technol 2006 12 43 49 10.1111/j.0909-725X.2006.00135.x 16420538

[B38] Shuster S Black MM McVitie E Influence of age and sex on skin thickness, skin collagen and density Br J Dermatol 1975 93 639 643 10.1111/j.1365-2133.1975.tb05113.x 1220811

[B39] El-Domyati M Attia S Saleh F Brown D Birk DE Gasparro F Ahmad H Uitto J Intrinsic aging vs. photoaging: a comparative histopathological, immunohistochemical, and ultrastructural study of skin Exp Dermatol 2002 11 398 405 10.1034/j.1600-0625.2002.110502.x 12366692

[B40] Robert C Lesty C Robert AM Aging of the skin - study of elastic fiber network modifications by computerized image-analysis Gerontology 1988 34 291 296 10.1159/000212969 2464530

[B41] Ghersetich I Lotti T Campanile G Grappone C Dini G Hyaluronic-acid in cutaneous intrinsic aging Int J Dermatol 1994 33 119 122 10.1111/j.1365-4362.1994.tb01540.x 8157393

[B42] Hayflick L Moorhead PS Serial cultiviation of human diploid cell strains Exp Cell Res 1961 25 585 621 10.1016/0014-4827(61)90192-6 13905658

[B43] Herbig U Ferreira M Condel L Carey D Sedivy JM Cellular senescence in aging primates Science 2006 311 1257 10.1126/science.1122446 16456035

[B44] Goyns MH Genes, telomeres and mammalian ageing Mech Ageing Dev 2002 123 791 799 10.1016/S0047-6374(01)00424-9 11869736

[B45] Sander CS Chang H Salzmann S Muller CSL Ekanayake-Mudiyanselage S Elsner P Thiele JJ Photoaging is associated with protein oxidation in human skin in vivo J Invest Dermatol 2002 118 618 625 10.1046/j.1523-1747.2002.01708.x 11918707

[B46] Gems D Doonan R Antioxidant defense and aging in C. elegans Is the oxidative damage theory of aging wrong? Cell Cycle 2009 8 1681 1687 10.4161/cc.8.11.8595 19411855

[B47] Fisher GJ Datta SC Talwar HS Wang ZQ Varani J Kang S Voorhees JJ Molecular basis of sun-induced premature skin ageing and retinoid antagonism Nature 1996 379 335 339 10.1038/379335a0 8552187

[B48] Lowe NJ Meyers DP Wieder JM Luftman D Borget T Lehman MD Johnson AW Scott IR Low-doses of repetitive ultraviolet A induce morphologic changes inhuman skin J Invest Dermatol 1995 105 739 743 10.1111/1523-1747.ep12325517 7490465

[B49] Hoffmann K Kaspar K Altmeyer P Gambichler T UV transmission measurements of small skin specimens with special quartz cuvettes Dermatology 2000 201 307 311 10.1159/000051543 11146339

[B50] Saarialho-Kere U Kerkela E Jeskanen L Hasan T Pierce R Starcher B Raudasoja R Ranki A Oikarinen A Vaalamo M Accumulation of matrilysin (MMP-7) and macrophage metalloelastase (MMP-12) in actinic damage J Invest Dermatol 1999 113 664 672 10.1046/j.1523-1747.1999.00731.x 10504457

[B51] Cavarra E Fimiani M Lungarella G Andreassi L de Santi M Mazzatenta C Ciccoli L UVA light stimulates the production of cathepsin G and elastase-like enzymes by dermal fibroblasts: A possible contribution to the remodeling of elastotic areas in sun-damaged skin Biol Chem 2002 383 199 206 11928814 10.1515/BC.2002.020

[B52] Chakraborti S Mandal M Das S Mandal A Chakraborti T Regulation of matrix metalloproteinases: An overview Mol Cell Biochem 2003 253 269 285 10.1023/A:1026028303196 14619979

[B53] Young AR Chromophores in human skin Phys Med Biol 1997 42 789 802 10.1088/0031-9155/42/5/004 9172259

[B54] Pattison DI Rahmanto AS Davies MJ Photo-oxidation of proteins Photochemical & Photobiological Sciences 2011 11 38 53 21858349 10.1039/c1pp05164d

[B55] Foote CS Definition of type-I and type-II photosensitized oxidation Photochem Photobiol 1991 54 659 10.1111/j.1751-1097.1991.tb02071.x 1798741

[B56] Davies MJ Reactive species formed on proteins exposed to singlet oxygen Photochem Photobiol Sci 2004 3 17 25 10.1039/b307576c 14743273

[B57] Wondrak GT Roberts MJ Cervantes-Laurean D Jacobson MK Jacobson EL Proteins of the extracellular matrix are sensitizers of photo-oxidative stress in human skin cells J Invest Dermatol 2003 121 578 586 10.1046/j.1523-1747.2003.12414.x 12925218

[B58] De Gruijl FR Rebel H Early Events in UV Carcinogenesis—DNA Damage, Target Cells and Mutant p53 Foci† Photochem Photobiol 2008 84 382 387 10.1111/j.1751-1097.2007.00275.x 18221455

[B59] Gibbs NK Norval M Urocanic Acid in the Skin: A Mixed Blessing? J Invest Dermatol 2011 131 14 17 10.1038/jid.2010.276 21157424

[B60] Rattan SIS Theories of biological aging: Genes, proteins, and free radicals Free Radic Res 2006 40 1230 1238 10.1080/10715760600911303 17090411

[B61] Sherratt MJ Tissue elasticity and the ageing elastic fibre Age 2009 31 305 325 10.1007/s11357-009-9103-6 19588272 PMC2813052

[B62] Verzijl N DeGroot J Thorpe SR Bank RA Shaw JN Lyons TJ Bijlsma JWJ Lafeber F Baynes JW TeKoppele JM Effect of collagen turnover on the accumulation of advanced glycation end products J Biol Chem 2000 275 39027 39031 10.1074/jbc.M006700200 10976109

[B63] Kerwin BA Remmele RL Protect from light: Photodegradation and protein biologics J Pharm Sci 2007 96 1468 1479 10.1002/jps.20815 17230445

[B64] Miles CA Sionkowska A Hulin SL Sims YJ Avery NC Bailey AJ Identification of an intermediate state in the helix-coil degradation of collagen by ultraviolet light J Biol Chem 2000 275 33014 33020 10893225 10.1074/jbc.M002346200

[B65] Cooper DR Davidson RJ Effect of Ultraviolet Irradiation on Soluble Collagen Biochem J 1965 97 139 147 16749094 10.1042/bj0970139 PMC1264553

[B66] Sionkowska A Kaminska A Thermal helix-coil transition in UV irradiated collagen from rat tail tendon Int J Biol Macromol 1999 24 337 340 10.1016/S0141-8130(99)00047-1 10408640

[B67] Sionkowska A Skopinska J Wisniewski M Leznicki A Fisz J Spectroscopic studies into the influence of UV radiation on elastin hydrolysates in water solution J Photochem Photobiol B Biol 2006 85 79 84 10.1016/j.jphotobiol.2006.05.005 16829118

[B68] Menter JM Patta AM Sayre RM Dowdy J Willis I Effect of UV irradiation on type I collagen fibril formation in neutral collagen solutions Photodermatol Photoimmunol Photomed 2001 17 114 120 10.1034/j.1600-0781.2001.170302.x 11419538

[B69] Menter JM Cornelison LM Cannick L Patta AM Dowdy JC Sayre RM Abukhalaf IK Silvestrov NS Willis I Effect of UV on the susceptibility of acid-soluble Skh-1 hairless mouse collagen to collagenase Photodermatol Photoimmunol Photomed 2003 19 28 34 10.1034/j.1600-0781.2003.00004.x 12713552

[B70] Du H Fuh RCA Li JZ Corkan LA Lindsey JS PhotochemCAD: A computer-aided design and research tool in photochemistry Photochem Photobiol 1998 68 141 142

[B71] LE Bensasson RV Truscott TG Excited States and Free Radicals in Biology and Medicine Contributions from Flash Photolysis and Pulse Radiolysis 1993 Oxford University Press Inc., New York

[B72] Kielty CM Sherratt MJ Shuttleworth CA Elastic fibres J Cell Sci 2002 115 2817 2828 12082143 10.1242/jcs.115.14.2817

[B73] Vieira-Silva S Rocha EPC An assessment of the impacts of molecular oxygen on the evolution of proteomes Mol Biol Evol 2008 25 1931 1942 10.1093/molbev/msn142 18579552 PMC2515869

[B74] Pravatà G Noto G Aricò M Increased SS bonds in chronic solar elastosis: a study with N-(7-dimethylamino-4-methyl-3-coumarinyl) maleimide (DACM) stain J Dermatol Sci 1994 7 14 23 10.1016/0923-1811(94)90017-5 8193080

